# Significant role of circRNA BBS9 in chronic obstructive pulmonary disease via miRNA-103a-3p/BCL2L13

**DOI:** 10.1186/s12890-023-02540-2

**Published:** 2023-07-13

**Authors:** Pujian Guo, Jing Lu, Yu Lei

**Affiliations:** Department of Pulmonary and Critical Care Medicine, Wuhan Fourth Hospital, Wuhan, 430030 China

**Keywords:** BCL2L13, Chronic obstructive pulmonary disease, circRNA BBS9, miRNA-103a-3p

## Abstract

**Background:**

Various studies have shown that circular RNA (circRNA) plays a pivotal role in chronic obstructive pulmonary disease (COPD). We aimed to determine the role of circRNA BBS9 in COPD progression.

**Methods:**

Real-time quantitative reverse transcription PCR (qRT-PCR) was performed to determine the levels and the linkages of circRNA BBS9, miRNA-103a-3p, and BCL2L13 in cigarette smoke extract (CSE)-treated human pulmonary microvascular endothelial cells (HPMECs). The target binding sites of circRNA BBS9 and miRNA-103a-3p were predicted using the starBase database, and the TargetScan algorithm was used to forecast the potential binding sites of BCL2L13 and miRNA-103a-3p, which were verified using a dual-luciferase reporter assay. An flow cytometry (FCM) assay was performed to determine the rate of apoptosis of HPMECs. Caspase3 activity was determined using a Caspase3 assay kit. The apoptosis-related protein bands were determined by western blotting.

**Results:**

The level of circRNA BBS9 increased in 1% CSE-induced cells, and silencing of circRNA BBS9 decreased the ratio of apoptotic cells among the 1% CSE-induced HPMECs. The results of dual-luciferase reporter assays showed that miRNA-103a-3p associates with circRNA BBS9. miRNA-103a-3p was downregulated in COPD, and upregulation of miRNA-103a-3p inhibited apoptosis in CSE-stimulated cells. Moreover, BCL2L13 was found to act downstream of miRNA-103a-3p. Silencing of miRNA-103a-3p reversed the inhibitory effect of circRNA BBS9-siRNA. The effects of the miRNA-103a-3p mimic were reversed by the BCL2L13-plasmid.

**Conclusion:**

circRNA BBS9 is involved in COPD development as it inhibits the functioning of miRNA-103a-3p. Our results suggest that circRNA BBS9 may act as a novel target for treating COPD.

**Supplementary Information:**

The online version contains supplementary material available at 10.1186/s12890-023-02540-2.

## Background

Chronic obstructive pulmonary disease (COPD), as the name of the disease suggests, is defined by airway obstruction [1]. According to global statistics for the year 2012, COPD (pulmonary obstruction) has become the third leading cause of death worldwide, and the number of deaths has gradually increased. In 2017, it ranked seventh among the top ten causes of death [2–4]. The two main types of COPD are emphysema and chronic bronchitis. Emphysema occurs at the ends of bronchioles and alveoli in the lungs, where the airways become blocked, trapping air, and causing the lungs to swell. Chronic bronchitis causes airway obstruction due to increased mucus secretion in the bronchi of the lungs, which is characterized by the appearance of white mucus bubbles in the sputum [5, 6]. There are several possible causes of pulmonary obstruction. The following are the common causes. Smoking: Smoking is the main cause of lung obstruction because smoking affects the function of cilia and phagocytes, causing repeated inflammation of the respiratory tract. Moreover, chronic exposure to secondhand smoke increases the risk of lung obstruction. Air pollution: Long-term exposure to excessive levels of particulate matter 2.5 (PM2.5) or various types of air pollutants, increases the risk of pulmonary obstruction. Dusty working environment: Long-term exposure to organic or inorganic substances such as coal, cotton wool, and silicon and dusty environments increase the risk of pulmonary obstruction [7, 8]. Further exploration of the pathogenesis of COPD will help in the development of novel treatment strategies with important practical significance. In this study, we identified novel biomarkers for the treatment of COPD.

Circular RNAs (circRNAs) are non-coding RNAs that form a circular conformation through covalent bonds. CircRNA is a single stranded covalently closed circular RNA molecule generated from a wide range of genomic regions, ranging from inter gene sequences, intron sequences, and coding sequences to 5’- or 3’ - untranslated sequences [9]. A series of new evidence suggests that although circRNAs can function as ncRNAs, such as in miRNA sponges, they can also encode proteins [10, 11]. Many reports have hinted that some circRNAs are involved in the pathogenesis of COPD. circRNA XPO1 plays a role in the pathogenesis of COPD by modulating TGF-β-activated kinase 1/MAP3K7 binding protein 3 (TAB3) [12]. circRNA TMEM30A is highly expressed in COPD [13]. circRNA BBS9 is a novel circRNA that may play a critical role in muscle aging [14]. Furthermore, circRNA BBS9 is also associated with osteoporosis [15]. Although the expression of circRNA BBS9 has been reported to be significantly increased in COPD models [16], the specifitic role and mechanism of its regulation in COPD remains unclear.

Researchers have found that some microRNAs (miRNAs) are involved in the development of several diseases [17, 18]. Recently, some reports have suggested that miRNAs play a critical role in COPD; for example, miRNA-486-5p is highly expressed in COPD [19]. miRNA-21 promotes COPD via the SATB1/S100A9/NF-κB axis [20]. Dexmedetomidine ameliorates COPD by regulating miRNA-146a [21]. MiR-103a-3p, which has been studied in various cancers [22, 23], is significantly down-regulated in the blood samples from pneumonia patients and LPS induced lung epithelial cells [24]. The function of miRNA-103a-3p and related mechanisms in COPD have not yet been elucidated.

B-cell lymphoma-2-like 13 (BCL2L13) is a BCL2-like protein belonging to the regulated cell death (RCD) protein family [25]. In a previous study, BCL2L13 was shown to participate in many human disease processes. For instance, circRNA 0062166 is regulated by BCL2L13 during cerebral ischemia-reperfusion [26]. The long non-coding RNA (lncRNA) SNHG15 protects against osteoarthritis by upregulating BCL2L13 [27]. However, the correlation between BCL2L13 expression and COPD has not yet been determined.

## Methods

### Cell cultures

Human pulmonary microvascular endothelial cells (HPMECs) and 293T cells used for the dual-luciferase assays were obtained from American Type Culture Collection (ATCC). The cells were cultured in dulbecco’s modified eagle medium (VivaCell, Shanghai, China) supplemented with 1% penicillin and streptomycin (VivaCell) and 15% fetal bovine serum (VivaCell) under conditions of 5% CO_2_ at 37 ºC.

HPMECs were stimulated using 1% cigarette smoke extract (CSE) for 24 h to generate a COPD cell model. Cigarettes were obtained from Jiangsu Zhongyan Industry Co., Ltd. (Nanjing, China). We used four lighted cigarettes and filtered the smoke using a 0.22-µm membrane; the filtered smoke was collected in 5 mL of serum-free medium.

### Bioinformatic analysis

The binding sites of miRNA-103a-3p on circRNA BBS9 were predicted using starBase 2.0 (http://starbase.sysu.edu.cn/index.php), and BCL2L13 fragments containing miRNA-103a-3p binding sites were predicted using TargetScan 7.2 (https://www.targetscan.org/vert_72/).

### Dual-relative luciferase reporter gene assays

The wild-type (WT) or mutated sequence of thw 3’ untranslated region (3’ UTR) of circRNA BBS9 and BCL2L13 sequence were incorporated into a pRL-TK vector (Tongyong, China) for analysis of luciferase activity. Subsequently, 293T cells were co-transfected with WT pRL-TK-circRNA BBS9 (or BCL2L13)-3′-UTR or mutated pRL-TK-circRNA BBS9 (or BCL2L13)-3′-UTR with mimic control and mimic of miRNA-103a-3p using jetPRIME, according to the manufacturer’s protocol (Polyplus, France). Renilla luciferase activity was measured 36 h after the infection using the reporter system (Promega, USA).

### Cell apoptosis assay

For analysis of apoptosis, 1 × 10^6^ CSE-stimulated HPMECs were cultured in 0.5 × 10^3^ µL of a solution containing 5 µL annexin V-fluorescein isothiocyanate (FITC) and 5 µL propidium iodide (PI) (Beyotime, Shanghai, China) at room temperature in dark for 40 min. The apoptotic rate was analyzed by flow cytometry (Beckman Coulter, USA) with the Kaluza Analysis (version 2.1.1.20653; Beckman Coulter, Inc.).

### Cell transfection

To down-regulate the expression of circRNA BBS9 in cells, the control-siRNA and circRNA BBS9-siRNA (circ BBS9-siRNA) were obtained from RIBOBIO (Guangzhou, China). For regulation of expression of miRNA-103a-3p, inhibitor of miRNA-103a-3p and inhibitor control (miRNA-103a-3p inhibitor: 5′-UUCUAUUCUUACCUCAAC-3′ and inhibitor control: 5′-CUACUAUUCGCGGUACUUAUC-3′, respectively) and mimic of miRNA-103a-3p (5′-AGCAGCAUUGUACAGGGCUAUGA-3′) and mimic control (5′-UGCUUGAUCGUAGCUGAUCGU-3′) were purchased from Sangon Biotech (Shanghai) Co., Ltd. (Shanghai, China). To overexpress BCL2L13, BCL2L13-plasmid was obtained from Santa Cruz Biotechnology. All sequences (2 µM control-siRNA, 2 µM circ BBS9-siRNA, 100 nM inhibitor control, 100 nM miR-103a-3p inhibitor, 100 nM mimic control, 100 nM miR-103a-3p mimic, 1 µg control-plasmid, or 1 µg BCL2L13-plasmid) were transfected into cells that had grown to 65% confluence using jetPRIME (Polyplus). After incubating the cells for 48 h at 37 ºC and 5% CO_2_, the transfected cells were harvested.

### Real-time quantitative reverse transcription PCR (qRT-PCR)

Total RNA was extracted from cells using ISOLATION TRIzol buffer® (Univ, Shanghai, China) following the manufacturer’s protocol, and cDNA was obtained by reverse transcribing RNA using the Titan One Tube RT-PCR Kit (Merck, USA). The miRNA levels were determined using TransScript® Probe SuperMix (Qsingke, China), and qRT-PCR was performed using PerfectStart® SYBR qPCR Mix (Transgene, Nanjing, China). The expression levels were estimated using the 2^−ΔΔCt^ methods. The primer sequences used for amplification of BBS9, miRNA-103a-3p, U6, BCL2L13, and β-actin are as follows: U6: 5′-CGCCCTGGCAGCGCAGTTATACTA-3′ (F) and 5′-GCTCGATGCTAGTGGTCCC-3′ (R); β-actin: 5′-CCATCGGGAGCTGCTGGATCC-3′ (F) and 5′-CGTCCGTGCGGCTGCGCTAGCCG-3′ (R); BBS9: 5′-GCCGTGAGCGGGAGCGGGCGGGCG-3′ (F) and 5′-GCGGTGCGGAAAGGGCCCTGC-3′ (R); miRNA-103a-3p: 5′-CGCTGCCTAGCTGCGGGTCG-3′ (F) and 5′-CCGTGCTACCGATCGTGGAGTC-3′ (R); BCL2L13: 5′-CGGCGGGTTCGTAGTCGGTCGC-3′ (F) and 5′-GGGCTGTGTGCCTGCGAGTC-3′ (R).

### Caspase3 assay

Caspase3 activity of HPMECs was analyzed using a Caspase3 activity assay kit (R&D Systems, USA). Briefly, the samples were digested using trypsin (VivaCell), harvested, and centrifuged at 1,000 rpm for 2 min. The cells in the supernatant were incubated with the Caspase3 activity buffer (R&D Systems) for 10 min. The supernatant was collected by centrifugation at 12,000 rpm for 15 min. Analysis was performed using a microplate reader (Bio-Rad).

### Western blot assay

HPMECs were lysed using radioimmune precipitation assay (RIPA) cleavage buffer (Fcmacs). Proteins were separated by 8% sodium dodecyl sulfate–polyacrylamide gel electrophoresis (SDSPAGE) and transferred to a polyvinylidene fluoride (PVDF) membrane (Whatman, USA). Phosphate buffer saline-Tween 20 (PBST) and 5% non-fat milk powder were used to seal the PVDF membrane. Then, the PVDF membrane was incubated overnight with the specific primary antibodies, including anti-cleaved-Caspase3 and anti-Caspase3, at 4 °C overnight. GAPDH was used as an endogenous control. On the second day, after incubation with the secondary antibodies, the bands were detected using an imaging system (Bio-Rad, USA), and the grayscale values of the proteins were analyzed using the ImageJ software version 1.8.0 (NIH, Bethesda, MD, USA).

### Statistical analysis

All experiments were repeated for at least three times. Mean ± standard deviation (SD) was used to represent data obtained from triplicate experiments. Student’s t-test was used to compare results obtained for two groups, and one-way analysis of variance (ANOVA) followed by Tukey’s test was used to compare results obtained for multiple groups. Statistical significance was set at P < 0.05.

## Results

### miRNA-103a-3p binds to circRNA BBS9

**Fig. 1:**
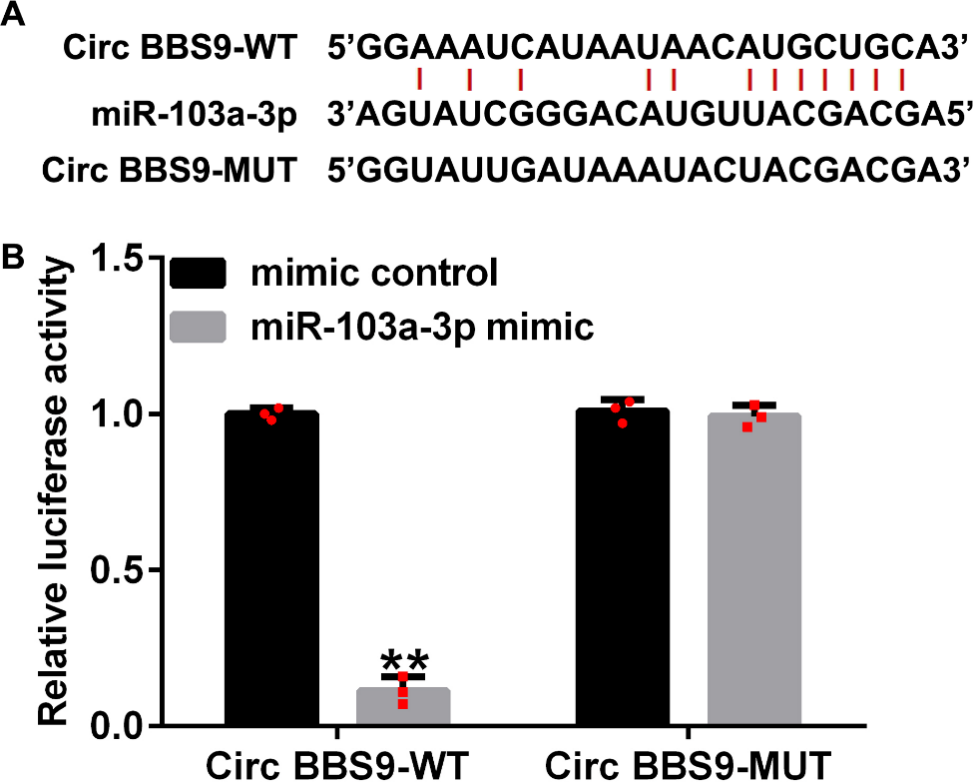
Circular RNA (circRNA) BBS9 targets miRNA-103a-3p. (**A**) The forecasted points of conjunction of miRNA-103a-3p and wild-type (WT) and mutated (Mut) BBS9. (**B**) The luciferase reporter activity observed upon co-transfection of cells with miRNA-103a-3p and WT and Mut BBS9. The data represent mean ± standard deviation (SD) obtained from results of experiments conducted in triplicates. **p < 0.01 vs. mimic control

The website starBase was used to predict the feasible binding points of circRNA BBS9 and miRNA-103a-3p. We discovered that miRNA-103a-3p contained putative circRNA BBS9 binding sites (Fig. 1A). As shown in Fig. 1B, the relative luciferase level of the circRNA BBS9-WT was notably reduced when the cells were co-cultured with a mimic of miRNA-103a-3p. When the potential binding sites were mutated, the miRNA-103a-3p mimic exhibited no effect. These results confirmed that miRNA-103a-3p was sponged by circRNA BBS9.

### CircRNA BBS9 is upregulated and miRNA-103a-3p is downregulated in COPD

**Fig. 2:**
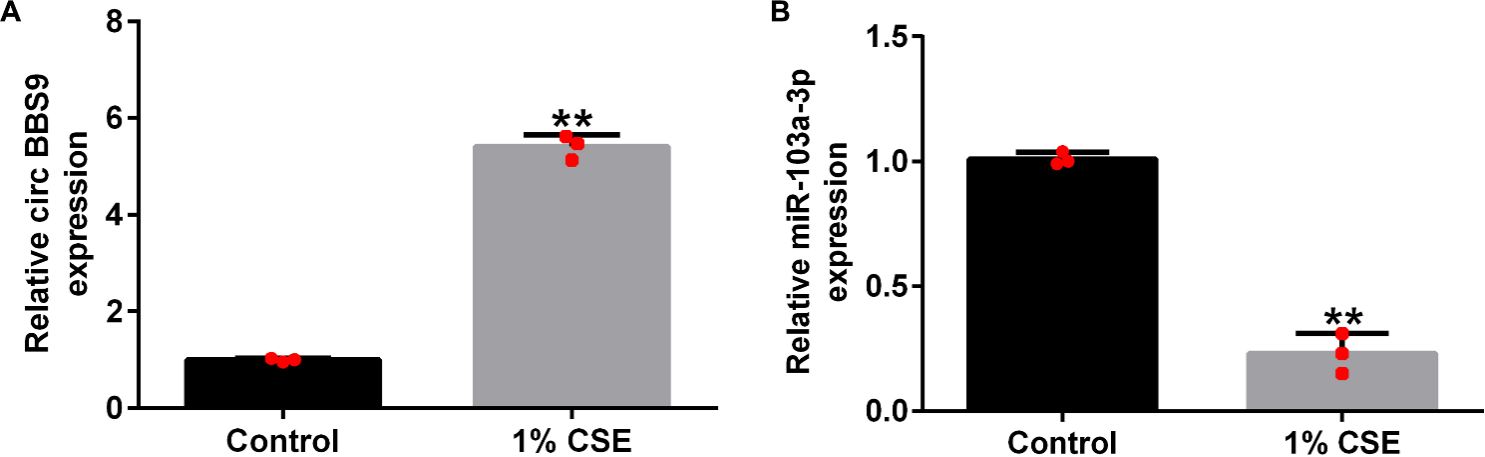
Levels of circRNA BBS9 and miRNA-103a-3p in chronic obstructive pulmonary disease (COPD). (**A**) Expression level of circRNA BBS9. (**B**) Expression level of miRNA-103a-3p. Data represent mean ± SD of experiments conducted in triplicates. **p < 0.01 vs. Control

BBS9 and miRNA-103a-3p levels were measured using qRT-PCR. The results indicated that the expression of circRNA BBS9 was upregulated in HPMECs treated with 1% CSE (Fig. 2A). Moreover, miRNA-103a-3p levels were reduced after stimulation of HPMECs with 1% CSE (Fig. 2B).

### Silencing of BBS9 suppresses apoptosis in 1% CSE-treated HPMECs via miRNA-103a-3p

**Fig. 3:**
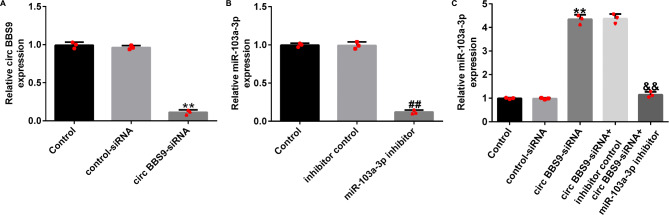
CircRNA BBS9 negatively regulates the expression of miRNA-103a-3p. (**A**) The efficiency of transfection with Circ BBS9-siRNA. (**B**) The efficiency of transfection with miRNA-103a-3p inhibitor. (**C**) The level of miRNA-103-3p in cells treated with Circ BBS9-siRNA and miRNA-103a-3p inhibitor. Data represent mean ± SD of experiments conducted in triplicates. **p < 0.01 vs. Control-siRNA; ## p < 0.01 vs. inhibitor control; *&&*p < 0.01 vs. Circ BBS9-siRNA + inhibitor control

To confirm the effects of BBS9 on COPD, we transfected HPMECs with circ BBS9-siRNA and miRNA-103a-3p inhibitors. Compared with that in the control-siRNA group, Circ BBS9-siRNA significantly reduced the expression level of circRNA BBS9 in HPMECs (Fig. 3A); compared with that in the inhibitor control group, the miRNA-103a-3p inhibitor significantly reduced the expression level of miRNA-103a-3p in HPMECs (Fig. 3B). Compared with that in the control-siRNA group, circ BBS9-siRNA significantly increased the expression level of miRNA-103a-3p in HPMECs co-transfected with the miRNA-103a-3p inhibitor (Fig. 3C).

**Fig. 4:**
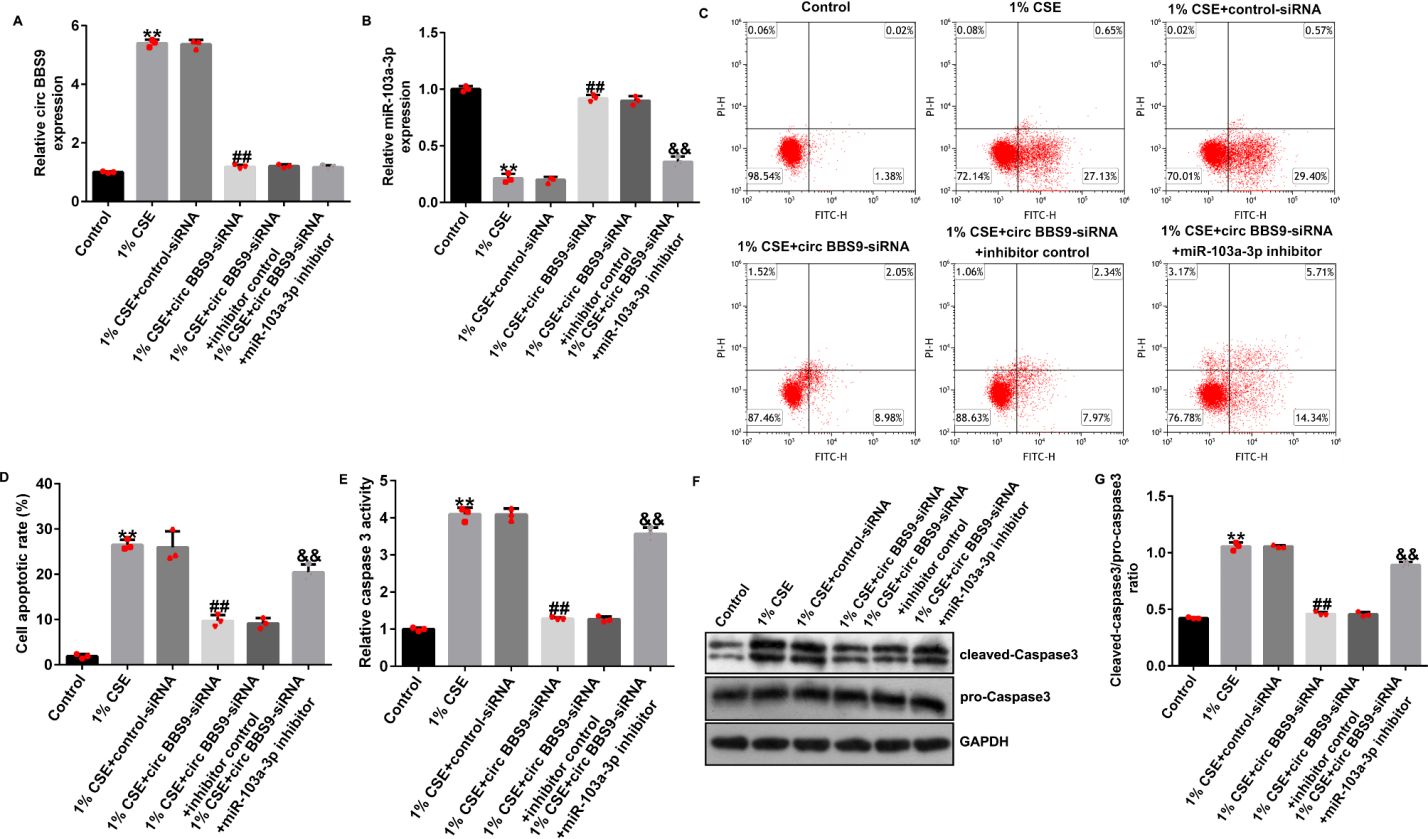
Impact of circRNA BBS9 inhibition in COPD cell model via targeting of miRNA-103-3p. (**A** and **B**) The expression levels of BBS9 and miRNA-103a-3p. (**C** and **D**) Apoptotic ratio of human pulmonary microvascular endothelial cells (HPMECs) was detected by FCM assay. (**E**) Activity of Caspase3 in COPD cell model. (**F** and **G**) The level of cleaved-Caspase3 was determined by western blotting. Data represent mean ± SD of experiments conducted in triplicates. **p < 0.01 vs. Control; ## p < 0.01 vs. 1% CSE + control-siRNA; *&&*p < 0.01 vs. 1% CSE + Circ BBS9-siRNA + inhibitor control

Compared with that in the control group, the expression level of circRNA BBS9 in the cells of the CSE group was significantly increased, and the expression level of miRNA-103a-3p was significantly decreased; compared with that in the CSE + control-siRNA group, the expression level of circRNA BBS9 in the cells of the CSE + circ BBS9-siRNA group was significantly decreased whereas the expression level of miRNA-103a-3p was significantly increased. The increase in expression level of miRNA-103a-3p that was induced by circ BBS9-siRNA was significantly inhibited upon co-transfection with the miRNA-103a-3p inhibitor (Fig. 4A and B). Compared with that in the control group, treatment with 1% CSE significantly induced apoptosis in HPMECs (Fig. 4C and 4D), increased the activity of Caspase3 (Fig. 4E), increased the protein expression level of cleaved-Caspase3 (Fig. 4F), and increased the ratio of cleaved-Caspase3/pro-Caspase3 (Fig. 4G); compared with that in the CSE + control-siRNA group, circ BBS9-siRNA significantly reduced the apoptosis of HPMECs (Fig. 4C and 4D), inhibited the activity of Caspase3 (Fig. 4E), reduced the protein expression level of cleaved-Caspase3 (Fig. 4F), and decreased the cleaved-Caspase3/pro-Caspase3 ratio (Fig. 4G); all these changes were significantly reversed upon co-transfection with the miRNA-103a-3p inhibitor.

### BCL2L13 acts downstream of miRNA-103a-3p

**Fig. 5:**
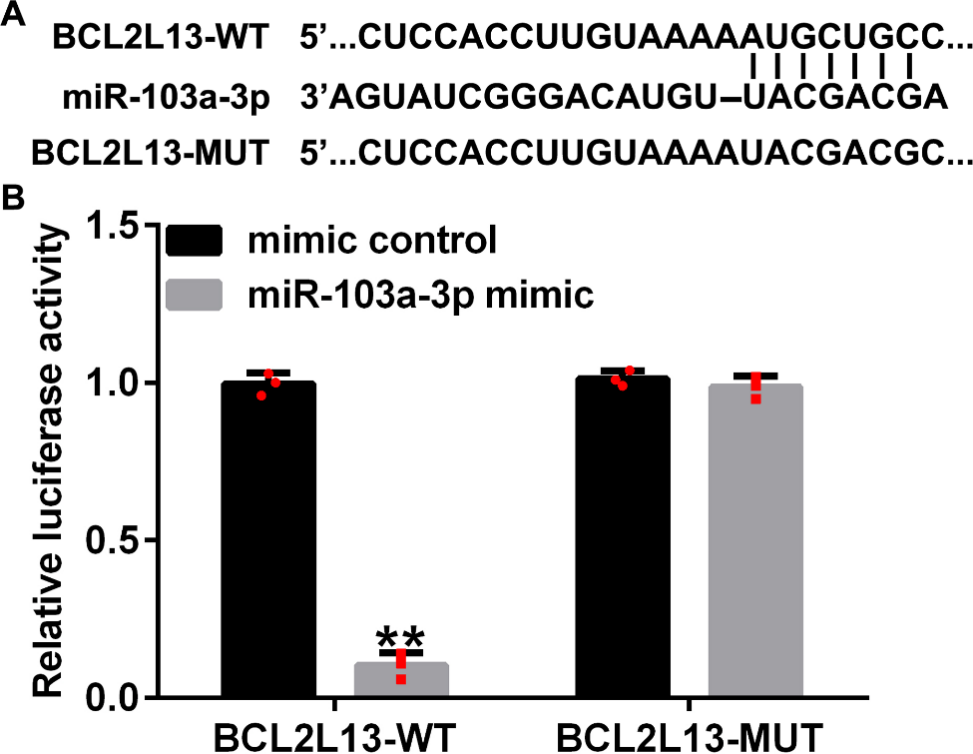
*BCL2L13* gene is a target of miRNA-103a-3p. (**A**) The binding sites of miRNA-103a-3p and BCL2L13 were predicted using TargetScan 7.0. (**B**) The dual-relative luciferase assay was performed to confirm the binding of miRNA-103a-3p with BCL2L13. Data represent mean ± SD of experiments conducted in triplicates. **p < 0.01 vs. mimic control

To identify the downstream target mRNA of miRNA-103a-3p, we used TargetScan 7.2 and found that BCL2L13 contained potential miRNA-103a-3p binding sites (Fig. 5A). Using the dual-relative luciferase method, we found that overexpression of miRNA-103a-3p led to a downregulation of the luciferase activity of the *BCL2L13-WT* reporter gene (Fig. 5B).

### miRNA-103a-3p is enriched in COPD and negatively regulates the expression of BCL2L13

**Fig. 6:**
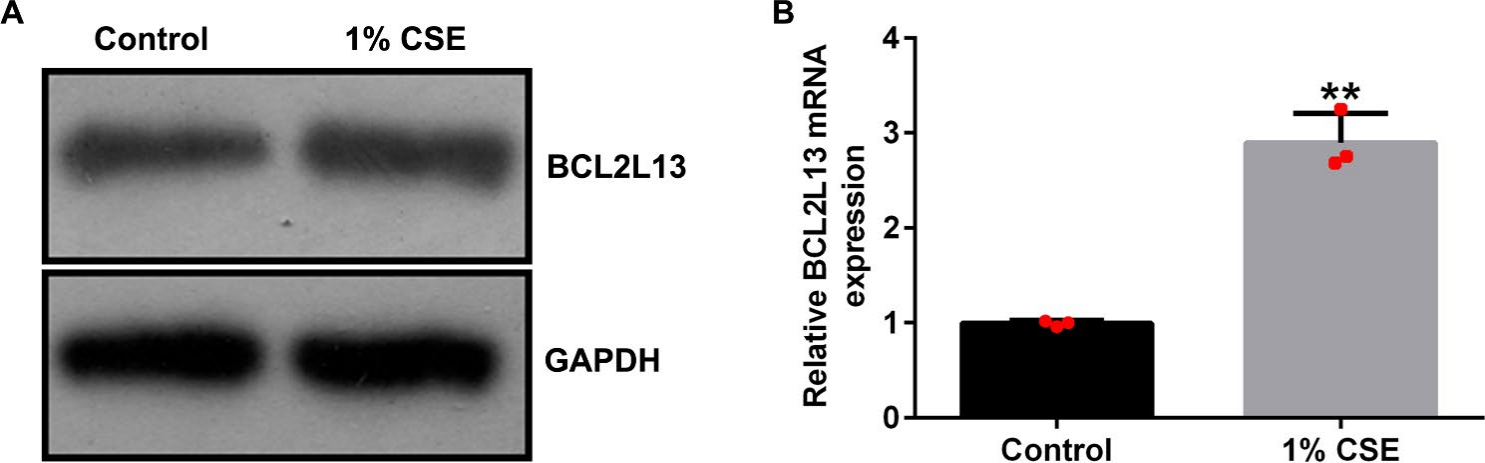
The level of BCL2L13 in COPD cell model. (**A**) The level of BCL2L13 mRNA in COPD cell model. (**B**) The level of BCL2L13 protein in COPD cell model. Data represent mean ± SD of experiments conducted in triplicates. **p < 0.01 vs. Control

**Fig. 7:**
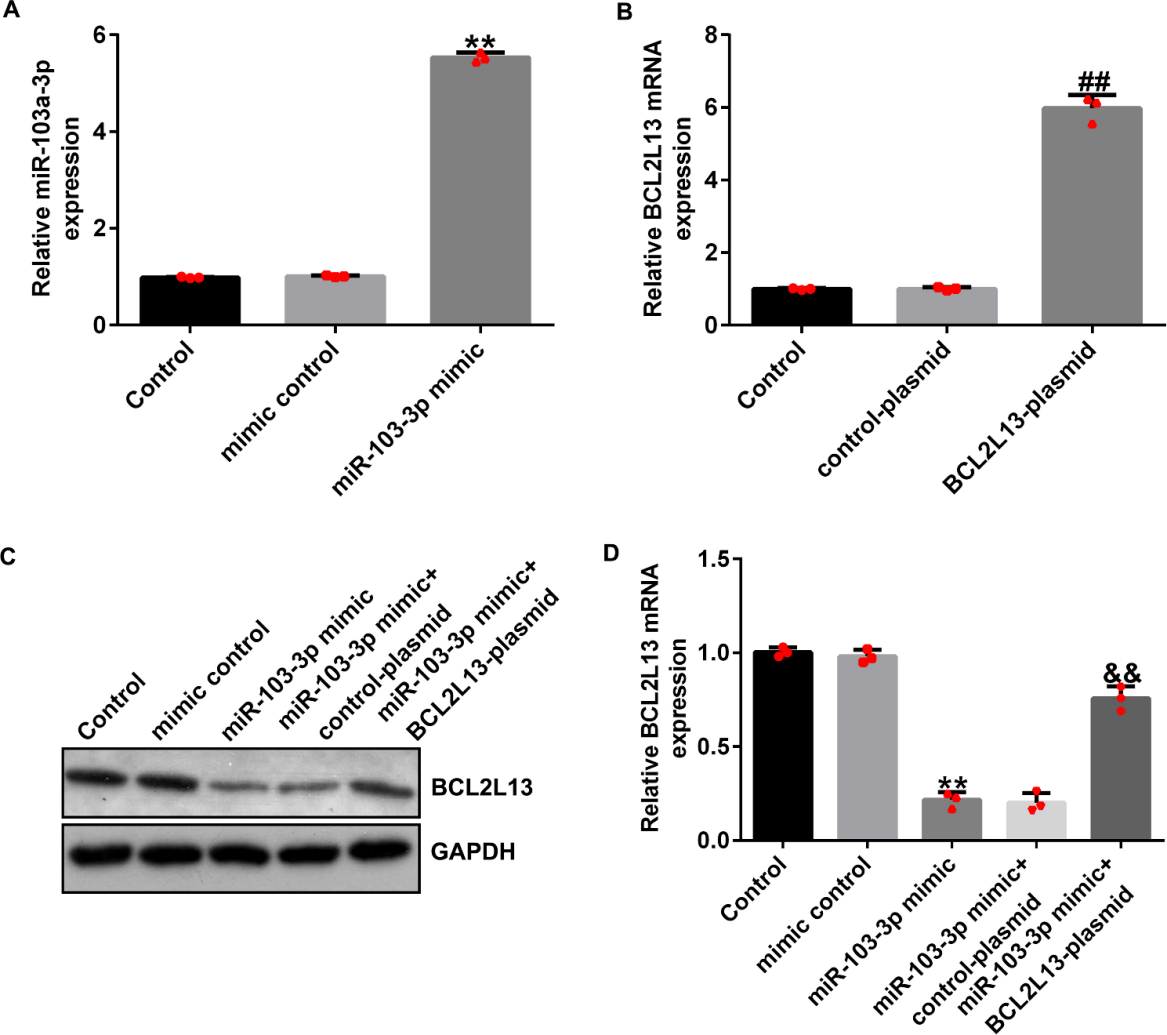
Effect of miRNA-103a-3p on BCL2L13 expression in HPMECs. (**A**) The transfection efficiency of miRNA-103a-3p mimic. (**B**) The transfection efficiency of BCL2L13-plasmid. (**C** and **D**) The level of BCL2L13 upon treatment of cells with miR-103-3p mimic and BCL2L13-plasmid. Data represent mean ± SD of experiments conducted in triplicates. **p < 0.01 vs. Mimic control; ## p < 0.01 vs. Control-plasmidl; *&&*p < 0.01 vs. miR-103-3p mimic + control-plasmid

In contrast to the findings obtained for the control group, the results of qRT-PCR and western blotting indicated that treatment with 1% CSE significantly increased the expression level of BCL2L13 in HPMECs (Fig. 6A and B). Compared with that in the mimic control group, the miRNA-103a-3p mimic significantly increased the expression level of miRNA-103a-3p in HPMECs (Fig. 7A), and the BCL2L13-plasmid significantly increased the expression level of BCL2L13 in HPMECs (Fig. 7B). The miRNA-103a-3p mimic significantly reduced the expression level of BCL2L13 in HPMECs, and this reduction was significantly reversed upon co-transfection with the BCL2L13-plasmid (Fig. 7C and 7D).

### miRNA-103a-3p attenuates CSE-induced apoptosis by inhibiting BCL2L13

**Fig. 8:**
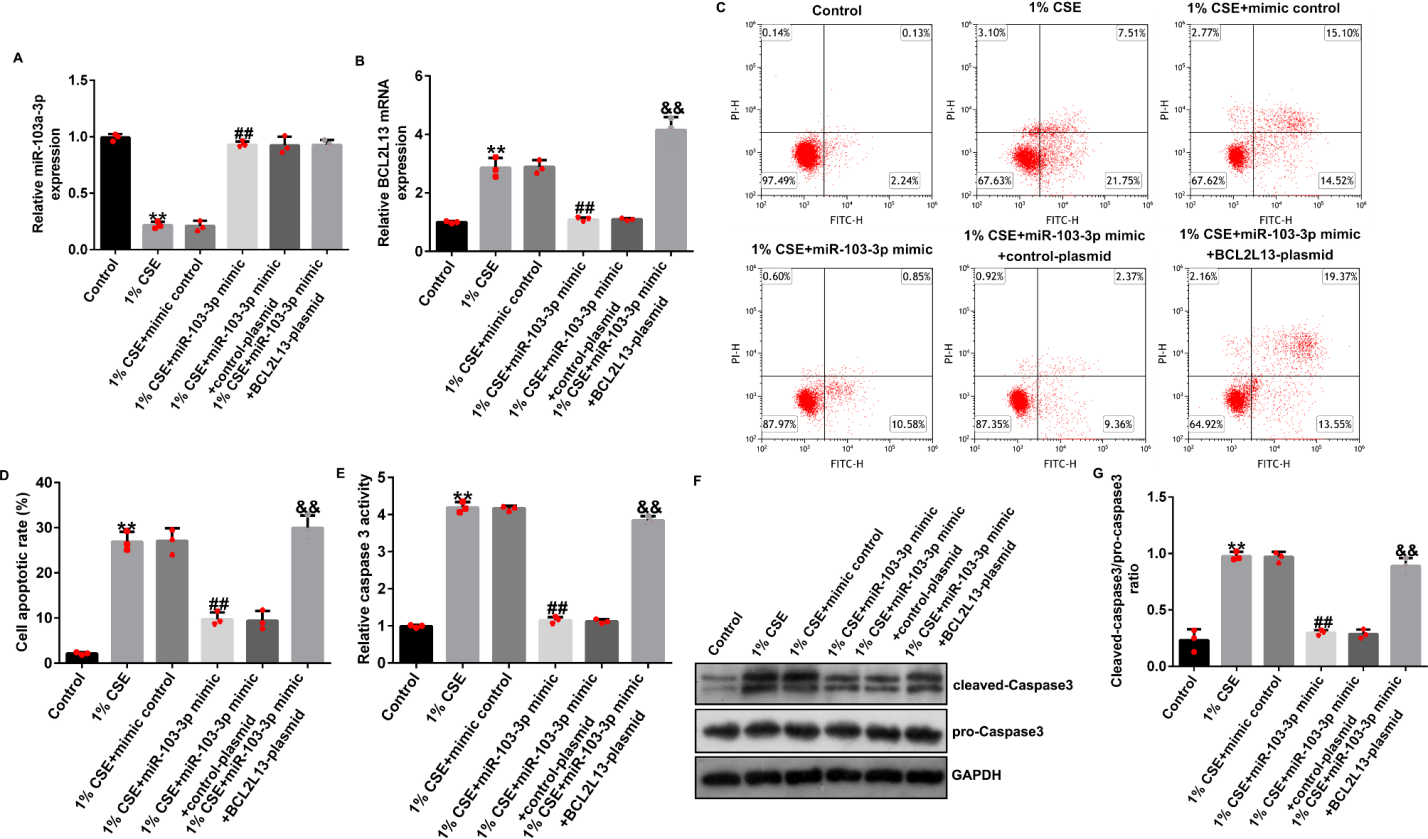
BCL2L13 reversed the effects of miRNA-103a-3p on 1% CSE-induced HPMECs. (**A** and **B**) The levels of miRNA-103a-3p and BCL2L13. (**C** and **D**) Apoptotic ratio of HPMECs was detected by FCM assay. (**E**) Activity of Caspase3 in COPD cell model. (**F** and **G**) The level of cleaved-Caspase3 was determined by western blotting. Data represent mean ± SD of experiments conducted in triplicates. **p < 0.01 vs. Control; ## p < 0.01 vs. 1% CSE + mimic control; *&&*p < 0.01 vs. 1% CSE + miR-103-3p mimic + control-plasmid

The HPMECs were transfected with the mimic control, the miRNA-103a-3p mimic, the miRNA-103a-3p mimic + control-plasmid, or the miRNA-103a-3p mimic + BCL2L13-plasmid for 24 h, and then the HPMECs were treated with 1% CSE for 24 h. The expression level of miRNA-103a-3p in the CSE group was significantly decreased, whereas the expression level of BCL2L13 was significantly increased; the expression level of miRNA-103a-3p was significantly increased and the expression level of BCL2L13 was significantly decreased when the cells were transfected with the miRNA-103a-3p mimic; the decrease in expression level of BCL2L13 caused by the miRNA-103a-3p mimic was significantly reversed upon co-transfection with the BCL2L13-plasmid (Fig. 8A and B).

Compared with that in the control group, treatment with 1% CSE significantly increased the apoptosis of HPMECs (Fig. 8C and 8D), increased the activity of Caspase3 (Fig. 8E), increased the protein expression level of cleaved-Caspase3 (Fig. 8F), and increased the ratio of cleaved-Caspase3/pro-Caspase3 (Fig. 8G). The miRNA-103a-3p mimic significantly inhibited apoptosis in HPMECs, decreased Caspase3 activity, decreased the cleaved-Caspase3 protein expression level, and decreased the cleaved-Caspase3/pro-Caspase3 ratio. These effects were significantly reversed upon co-transfection with the BCL2L13-plasmid.

## Discussion

The current situation of COPD in China is serious, and the mortality rate is high. COPD is a type of chronic bronchitis or emphysema characterized by airflow obstruction. COPD not only seriously endangers our health but is also very easy to ignore and can cause many diseases such as pulmonary heart disease, heart failure, respiratory hypoxia, and dysfunction of body organs [28].

The results of a large-scale population study conducted by the “China Adult Lung Health Study” published in “The Lancet” in April 2018 showed that the number of patients with COPD in China has reached 100 million, and the prevalence of COPD in people over 40 years of age is 13.7%. Moreover, the prevalence increased by 67% in just 13 years (2002–2015) [29].

Recently, circRNAs have been shown to play important roles in COPD. Many circRNAs have been described as biomarkers of COPD; circRNA-OSBPL2 is overexpressed in COPD and regulates COPD via miRNA-193a-5p and bromodomain-containing protein 4 (BRD4) [30]. CircRNA-FOXO3 was shown to treat smoke-related COPD in mice models [31]. Moreover, the inhibition of circRNA-XPO1 reduced inflammation in COPD-affected tissues [12]. Wang et al. reported that the knockdown of circRNA-ANKRD11 protected HPMECs from the effects of stimulation by CSE [32]. However, few studies have investigated the role of circRNA BBS9 in COPD. Consistent with previous research, [16], we found that the circRNA BBS9 was enriched in COPD, which may help in increasing our understanding of the pathological mechanism of COPD.

Several miRNAs are involved in the pathogenesis of COPD. In a previous report, it was shown that miRNA-145 and miRNA-338 are involved in COPD [33]. In another study, it was demonstrated that miRNA-221-3p accelerates the inflammatory response in COPD [34]. miRNA-130a assisted in development of smoke-induced COPD [35]. MiR-103a-3p has been reported to be low expressed in blood samples of pneumonia patients, and overexpression of miR-103a-3p weakens LPS induced pulmonary epithelial inflammatory response [24]. Here, we showed that miRNA-103a-3p was the downstream target of circRNA BBS9, and it was down-regulated by CSE treatment. Endothelial cells, as the fundamental unit of blood vessels, play an important role in physiological metabolism. Abnormal cell apoptosis is an important factor leading to the destruction of COPD lung tissue [36, 37]. The apoptosis of human pulmonary microvascular endothelial cells induced by CSE has been widely used to study COPD in vitro [38, 39]. The findings of current study showed that circRNA BBS9 gene silencing alleviates CSE induced human pulmonary microvascular endothelial cell apoptosis by increasing miR-103a-3p expression.

BCL2L13 is an important gene that is involved in development of several human diseases such as papillary renal cell carcinoma [40], cerebral ischemia/reperfusion injury [41], and glioblastoma [42]. In this study, BCL2L13 was identified as a target of miR-103a-3p, and BCL2L13 mRNA and protein are significantly increased by CSE induction, suggesting the association with COPD progression. Further analysis indicated that miR-103a-3p reduced CSE induced human pulmonary microvascular endothelial cell apoptosis by the down-regulation of BCL2L13 expression.

Taken together, the data of this study revealed that circRNA BBS9 inhibition inhibited HPMECs apoptosis induced by CSE through the regulation of the miR-103a-3p/BCL2L13 axis (Supplementary Fig. 1), suggesting a key role in COPD. However, this study still has some limitations. This study did not clarify the expression of circRNA BBS9/miR-103a-3p in COPD patients and its correlation with clinical pathological parameters. Besides, the role and mechanism of circRNA BBS9/miR-103a-3p in COPD should be validated in animal models. In the future, we will conduct in-depth research on these issues.

## Conclusions

Our study demonstrated that silencing of circRNA BBS9 represses apoptosis in HPMECs induced by CSE by regulating the miR-103a-3p/BCL2L13 axis The results suggest that circRNA BBS9 could provide a novel approach for the treatment of COPD.

## Electronic supplementary material

Below is the link to the electronic supplementary material.

Supplementary Material 1

Supplementary Material 2

## Data Availability

The datasets used and/or analyzed during the present study are available from the corresponding author on reasonable request.
