# Supplementary material for: Significant role of circRNA BBS9 in chronic obstructive pulmonary disease via miRNA-103a-3p/BCL2L13
Source: BMC Pulm Med. 2023 Jul 13;23:257. doi: 10.1186/s12890-023-02540-2 (PMC10347774; doi:10.1186/s12890-023-02540-2)
Supplement: Supplementary file 1 — Supplementary Material 1 [file 12890_2023_2540_MOESM1_ESM.docx]

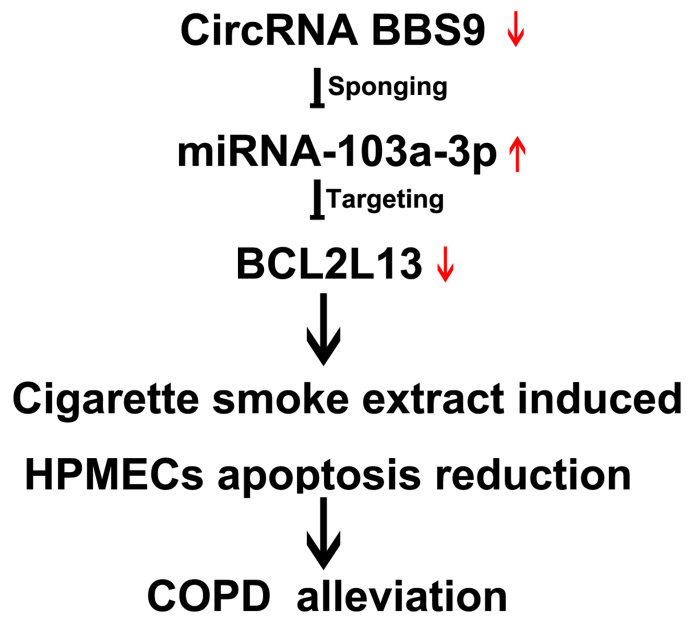


**Supplementary Figure 1. The graphical abstract of the manuscript.**

The regulatory pathway of circRNA BBS9 on apoptosis of HPMECs.
