# Supplementary material for: Significant role of circRNA BBS9 in chronic obstructive pulmonary disease via miRNA-103a-3p/BCL2L13
Source: BMC Pulm Med. 2023 Jul 13;23:257. doi: 10.1186/s12890-023-02540-2 (PMC10347774; doi:10.1186/s12890-023-02540-2)
Supplement: Supplementary file 2 — Supplementary Material 2 [file 12890_2023_2540_MOESM2_ESM.docx]

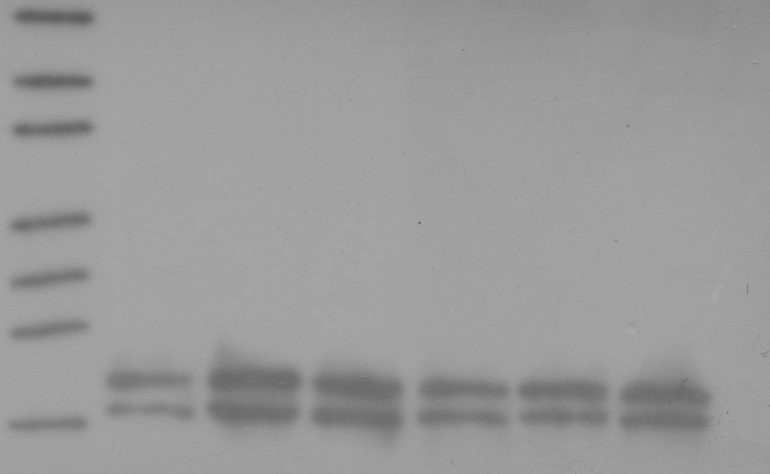

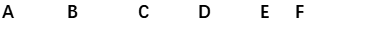


Cleaved Caspase3（Figure 4）19;17kDa

Marker


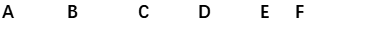

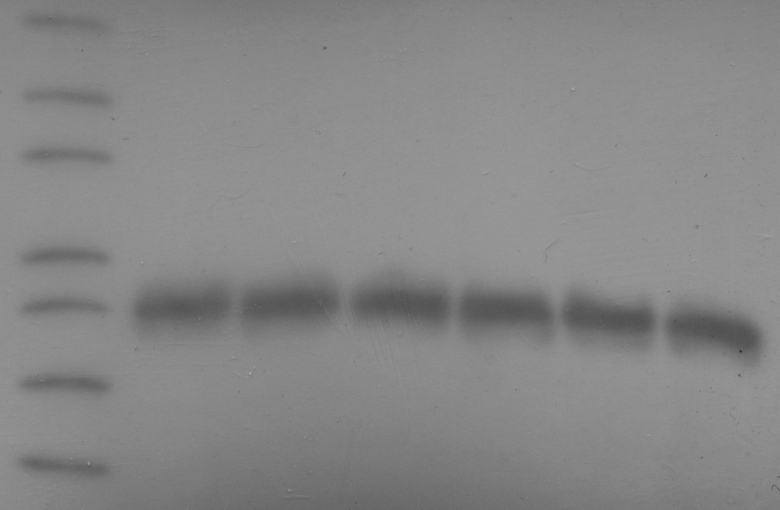


Pro-Caspase3 (Figure 4) 35kDa

Marker


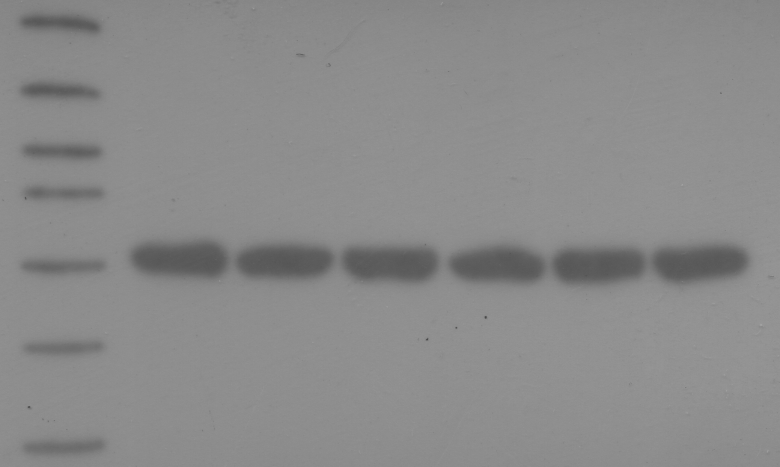

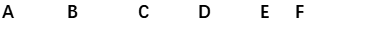


Marker

GAPDH （Figure 4）36kDa


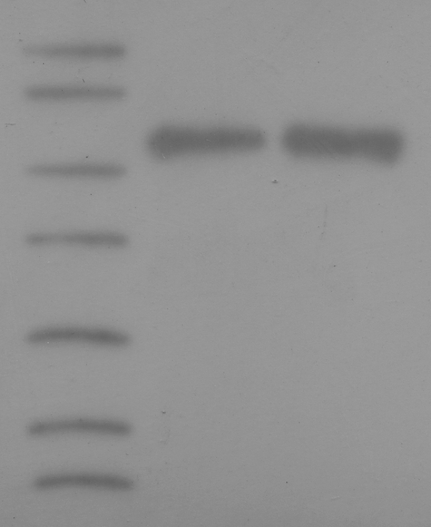

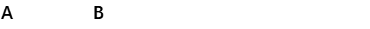


Marker

BCL2L13（Figure 6）

85kDa


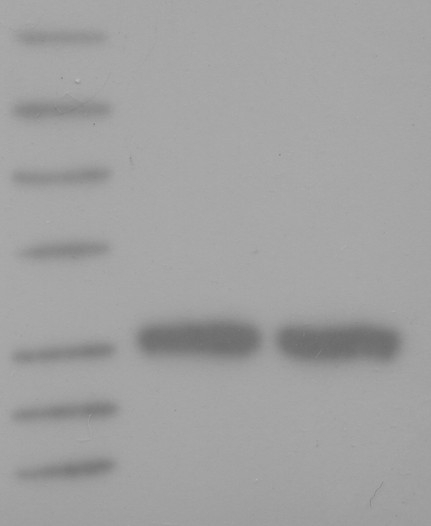

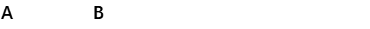


GAPDH （Figure 6）36kDa

Marker


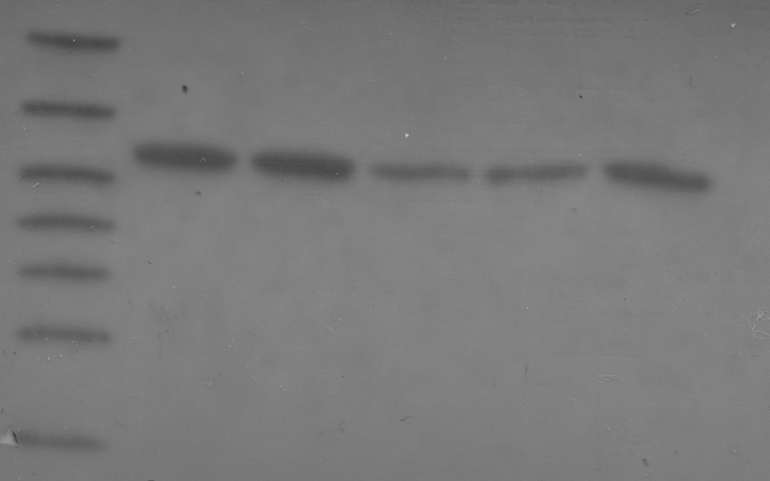

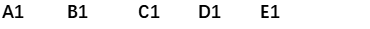


BCL2L13（Figure 7）85kDa

Marker


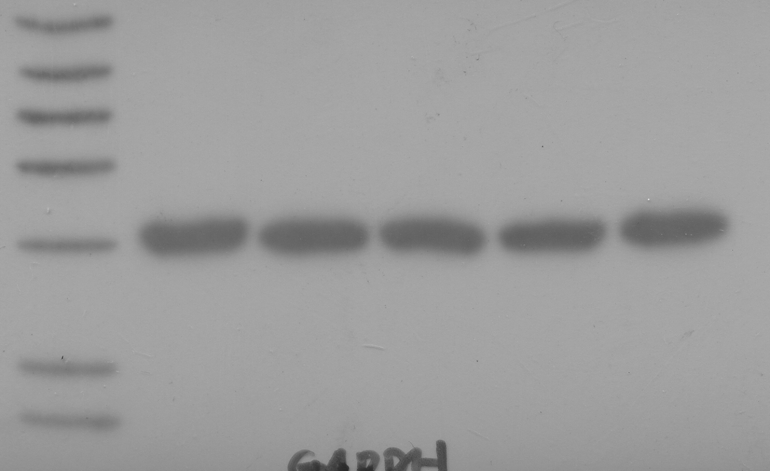

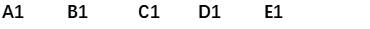


GAPDH （Figure 7）36kDa

Marker


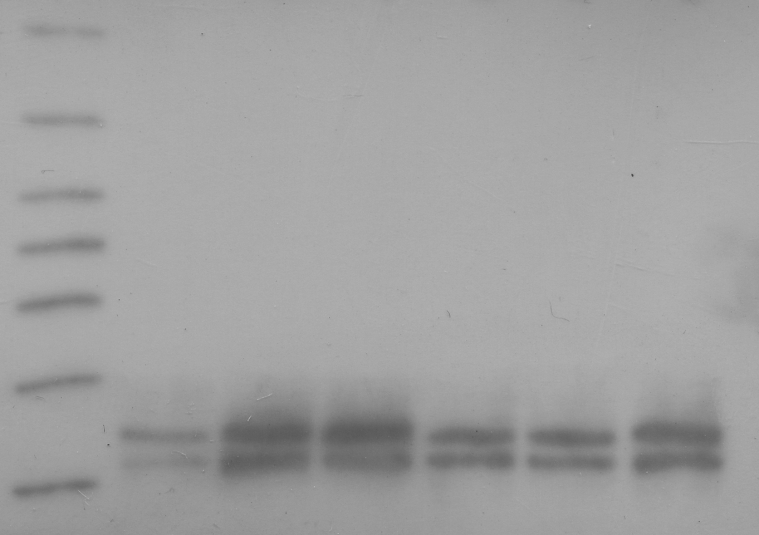

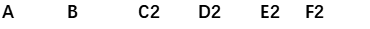


Cleaved Caspase3（Figure 8）19;17kDa

Marker


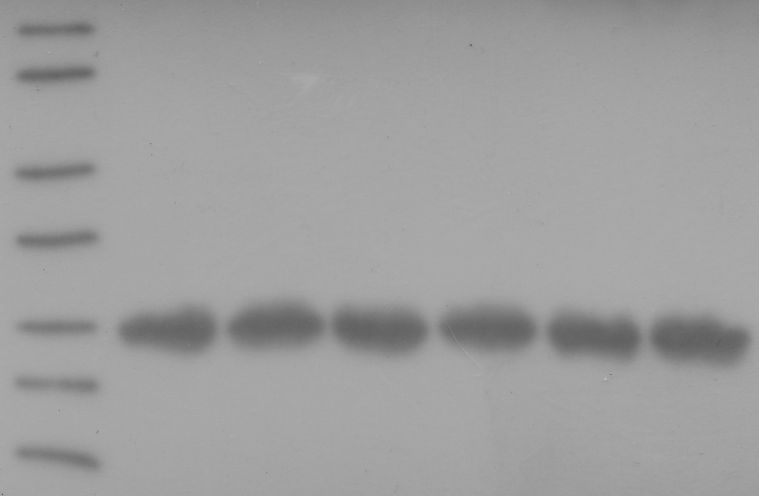

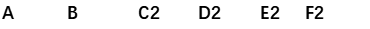


Pro-Caspase3 (Figure 8) 35kDa

Marker


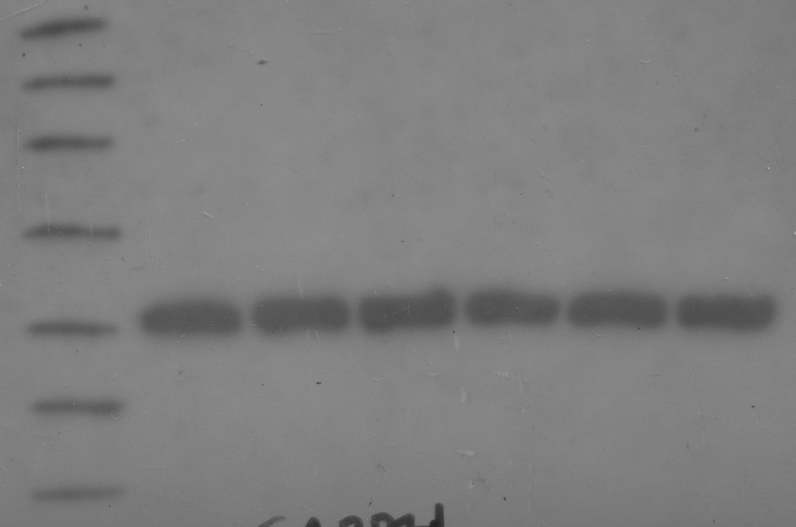

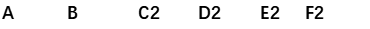


GAPDH （Figure 8）36kDa

Marker
